# Long-term outcomes of a dual-mobility cup and cementless triple-taper femoral stem combination in total hip replacement: a multicenter retrospective analysis

**DOI:** 10.1186/s13018-019-1436-y

**Published:** 2019-11-21

**Authors:** Alain Cypres, Arnaud Fiquet, Philippe Girardin, David Fitch, Philippe Bauchu, Olivier Bonnard, Daniel Noyer, Christophe Roy

**Affiliations:** 1 Clinique du Renaison, 75 Rue Général Giraud, 42300 Roanne, France; 2 Infirmerie Protestante, Caluire et Cuire, France; 3 Hôpital de Montbrison, Montbrison, France; 4 Smith & Nephew, Cordova, TN USA; 5 GHM les portes du Sud, Venissieux, France; 6 Clinique du Tonkin, Villeurbanne, France; 7 Hôpital de Bourgoin-Jallieu, Bourgoin-Jallieu, France; 8 Group GILES, Chatuzange, France

**Keywords:** Total hip replacement, Dual-mobility cup, Triple-taper stem, Survivorship, Outcomes, Polarstem, Polarcup

## Abstract

**Background:**

The separate design concepts of dual-mobility cups and triple-taper femoral stems were developed to improve survivorship following total hip replacement (THR) by reducing instability/dislocation and enabling enhanced fixation. Successful outcomes at over two decades have been reported with earlier-generation devices based on these concepts. The current study aimed to provide the first long-term results with a unique pairing of later-generation dual-mobility cup and triple-taper cementless femoral stem after a decade of use in patients undergoing THR.

**Methods:**

In this retrospective analysis, records were reviewed for all subjects implanted with this dual-mobility cup/cementless femoral stem combination at three centers between 2002 and 2005. Any subject who had not already had follow-up visit beyond 10 years, was not previously revised, and still living were invited for a single follow-up visit consisting of Merle d’Aubgine Scores, the Western Ontario and McMaster Universities Osteoarthritis (WOMAC) index, and standard radiographs.

**Results:**

There were 244 THRs available for analysis. At a mean follow-up of 11.9 years, the Kaplan-Meier survivorship (endpoint: revision for any reason) was 99.1% (95% CI, 97.6–99.7) for the stem and 95.9% (95% CI, 93.1–97.6) for the cup. Merle d’Aubigne Scores were significantly improved from baseline and WOMAC scores were in the satisfactory range at the final follow-up. Radiographic analysis revealed no cases of stem subsidence, no cases of bone hypertrophy, 1 (0.4%) case of bone atrophy, and 3 (1.2%) cases of osteolysis around the stem. No subjects had radiolucent lines greater than 1 mm in any femoral Gruen zone. Evidence of cup migration was seen in 1 (0.4%) subject and 1 (0.4%) subject had evidence of osteolysis that was seen in Gruen zones I, II, IV, and V.

**Conclusions:**

This combination of a later-generation dual-mobility cup and cementless triple-taper stem was associated with excellent survivorship and satisfactory functional outcomes at over 10 years follow-up.

**Trial registration:**

ClinicalTrials.gov, NCT02648152. Date of registration: January 6, 2016. Retrospectively registered.

## Introduction

Successful outcomes are routinely observed for total hip replacement (THR), with data from both case series and joint registries indicating that all-cause survivorship of > 85% can be achieved 15 years after primary surgery [1]. Nonetheless, there is a continual drive to improve upon these outcomes by addressing the underlying causes of component revision, a major source of which is instability/dislocation [2].

Among the design concepts created to provide increased stability after THR are dual-mobility cups, first introduced in the 1970s by Bousquet and colleagues [3]. Dual-mobility cups combine the foundational principles of low friction, which includes a mobile polyethylene liner locked on a femoral head articulating in a smooth metallic acetabular shell. This double articulating bearing with a large head-to-neck ratio results in enhanced joint mobility before the outer edges of the liner impinges against the femoral neck [4]. Early dual-mobility cups proved adept at decreasing dislocation rates, particularly among elderly patients at high risk for this outcome [5]. Similarly encouraging results have been reported with later-generation dual-mobility cups [6–11]. The continued positive safety and performance observed with these designs has extended their use into younger, more active patients [12], as well as the obese [13].

Triple-tapered femoral stems were introduced as a design concept in the 1980s, with a goal of providing enhanced fixation, reduced subsidence, and improved loading of the proximal femoral neck [14, 15]. Cementless triple-tapered femoral stem designs have well-documented clinical success over two decades of use [16–20].

The current study was undertaken to determine the long-term survivorship and clinical outcomes of a THR combination consisting of newer-generation cementless dual-mobility cup and cementless triple-tapered stem at a minimum of 10 years follow-up. Although both devices build upon established concepts, this represents the first published report of long-term outcomes with this specific combination. It was hypothesized that these components would lead to revision rates beneath the threshold of 5% at 10 years established by the Orthopaedic Data Evaluation Panel (ODEP) in the UK [21] as a marker of a successful THR.

## Materials and methods

The study was a multicenter retrospective analysis of all the subjects who underwent primary THR with the POLAR System, comprised of a cementless triple-tapered stem (POLARSTEM™; Smith & Nephew Orthopaedics AG, Baar, Switzerland) and cementless dual-mobility cup (POLARCUP™; Smith & Nephew Orthopaedics AG, Baar, Switzerland), at three centers between 2002 and 2005. All non-revised and non-deceased subjects who were at least 18 years old at the time of implantation and that had received the subject dual-mobility cup and femoral stem unilaterally or bilaterally for osteoarthritis, degenerative joint disease, traumatic events, or inflammatory/rheumatoid processes were invited to return for a follow-up visit at a minimum of 10 years after their original surgery. Subjects were not invited back for a visit if they already had a clinic visit beyond 10 years follow-up, had a medical or health condition which could impair their ability or willingness to comply with the study, or if they refused to sign the informed consent document.

The study’s primary objective was to evaluate the long-term survivorship for the dual-mobility cup and cementless triple-taper femoral stem after a decade of use following THR. The secondary objective of the study was to assess the safety and effectiveness of this combination in terms of radiographic and clinical performance.

Medical records were accessed and analyzed for subjects that already had the 10-year follow-up, and in case of missing information, subjects were invited for 10-year plus visit. Subjects returning for the minimum 10-year follow-up visit were assessed using the Western Ontario and McMaster Universities Osteoarthritis (WOMAC) index, Merle D’Aubigne Score, standard radiographs, and evaluated for any complications or revisions. Baseline preoperative Merle D’Aubigne Scores were collected from the medical records when available. Subjects who declined to return, or were previously revised, or were deceased had limited data collected from their medical records. This data included gender, age, study device, and any complications or revisions.

### Device description

**Fig. 1:**
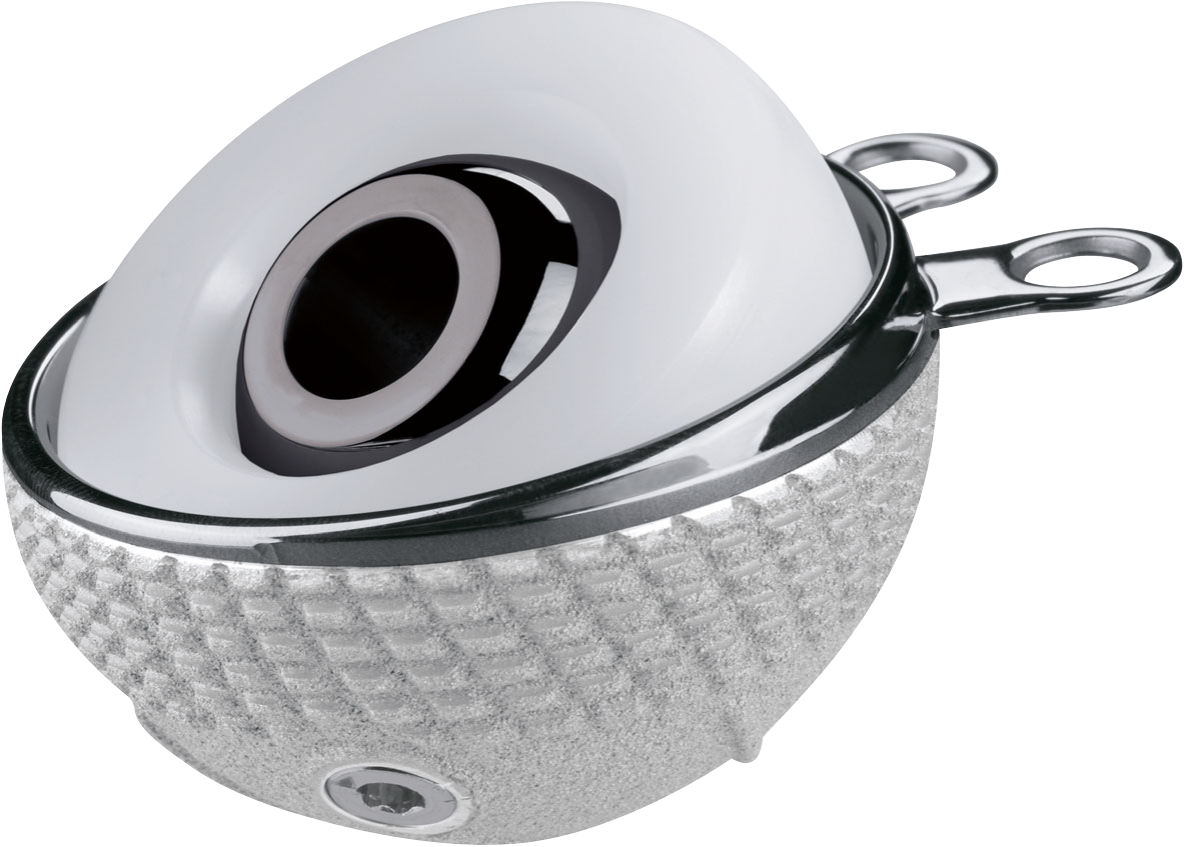
Dual-mobility Polarcup system

**Fig. 2:**
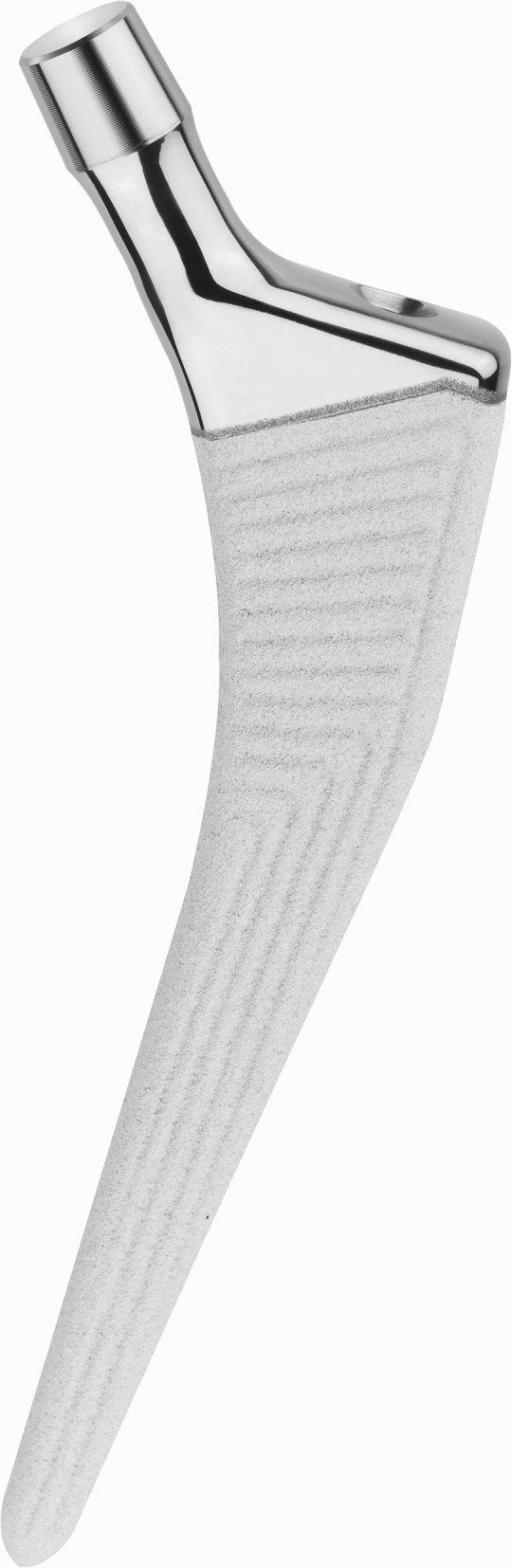
Side view of the triple-taper Polarstem

All subjects received the dual-mobility system with cup size ranging from 43 to 63 mm (Fig. 1). This system features an acetabular liner conventional or highly cross-linked polyethylene inserts articulating with either ceramicised metal, ceramic, or cobalt chrome femoral heads. Additionally, patients received the cementless femoral stem (Fig. 2), which was lateral in 39 subjects and standard in 205. This stem is a triple-tapered design manufactured from a Ti-6Al-4 V alloy. It features a 180-μm titanium plasma spray that is coated with 50 μm of hydroxyapatite. Femoral head size included 22 mm and 28 mm and the neck of the stem is mirror-polished with a 12/14 taper. It is shorter than similar triple-taper designs, which may make it easier to use with muscle-sparing surgical techniques.

### Statistical considerations

Descriptive statistics were used to summarize demographics, WOMAC Scores, Merle D’Aubigne Scores, radiographic outcomes, and complications. Kaplan-Meier analysis was used to evaluate component survivorship.

## Results

There were 502 THRs implanted during the period of interest that satisfied the inclusion/exclusion criteria, of which 106 refused to return and 152 were deceased, leaving 244 who agreed to return for a follow-up visit (Table 1).

**Table 1:** Demographics for 224 subjects (244 hips) available for the 10-year follow-up visit

Variable	*N*
Gender	
Female	97 (43.3%)
Male	127 (56.7%)
Hip side	
Left	111 (45.5%)
Right	133 (54.5%)
Mean subject age at surgery, years (range)	63.8 (29–82)
Mean body mass index at surgery (range)	27.6 (15.6–41.4)
Primary diagnosis for surgery	
Avascular necrosis	15 (6.1%)
Dysplasia	8 (3.3%)
Femoral neck fracture	2 (0.8%)
Missing	1 (0.4%)
Osteoarthritis	209 (85.7%)
Other	2 (0.8%)
Post-traumatic arthritis	2 (0.8%)
Rheumatoid arthritis	5 (2.0%)

At a mean follow-up time of 11.9 years (range, 10.2–14.3 years), the Kaplan-Meier survivorship was 99.1% (95% CI, 97.6–99.7) with revision of the femoral stem for any reason as the endpoint and 95.9% (95% CI, 93.1–97.6) with revision of the acetabular component for any reason as the endpoint. There were 4 revisions of the femoral stem, all of which were required due to fractures that occurred at 14 days and at 1.1, 24.2, and 61.2 months. There were 17 acetabular component revisions in 15 subjects. In 14 subjects, this was due to aseptic loosening, with revisions occurring at 24.2, 35.4, 80.0, 80.4, 91.3, 96.5, 97.0, 99.4, 112.7, 117.2, 118.7, 121.7, 125.6, and 133.7 months. In the remaining subject, the revision was secondary to a fracture of the femur and occurred at 70.9 months.

Intraoperative and early postoperative complications included 2 (0.8%) superficial infections, 2 (0.8%) hematomas, 1 deep infection (0.4%), 1 (0.4%) femoral fissure, and 1 (0.4%) case of delirium tremens.

Radiographic analysis revealed no cases of stem subsidence, no cases of bone hypertrophy, 1 (0.4%) case of bone atrophy, and 3 (1.2%) cases of osteolysis around the stem. The case of bone atrophy occurred in a subject with a history of developmental dysplasia and who had multiple acetabular revisions prior to the long-term follow-up visit. No subjects had radiolucent lines greater than 1 mm in any femoral Gruen zone. Evidence of cup migration was seen in 1 (0.4%) subject and 1 (0.4%) subject had evidence of osteolysis that was seen in Gruen zones I, II, IV, and V.

At the final follow-up, the mean WOMAC total, pain, stiffness, and function scores were 13.9 (range, 0–75), 2.3 (range, 0–14), 1.1 (range, 0–6), and 10.7 (range, 0–56), respectively. Mean Merle D’Aubigne Scores improved from 9.9 (range, 6–15) to 17.0 (range, 10–18).

## Discussion

This group had previously reported low revision for this cohort at 3 years follow-up [22]; however, the current study represents the first long-term analysis of the survivorship of this cup/stem combination. The results confirm that excellent survivorship can be achieved with this dual-mobility cup/cementless triple-taper stem combination after a decade in situ. At nearly 12 years follow-up, the survivorship for the cup was 95.9% and for the femoral stem was 99.1%. Therefore, revision rates for both components were beneath the threshold of 5% revision rate at 10 years used by ODEP [21], which is a widely accepted benchmark for judging the long-term performance of a THR device.

Cementless THR is the most commonly used method of fixation [23]. The key goals for cementless THR are achieving durable primary mechanical stability and osteointegration between implant and bone [24]. The design concepts utilized in this study offer several possible mechanisms for meeting these objectives.

Dual-mobility cups are thought to lower the risk of dislocation by minimizing prosthetic neck impingement and increasing range of motion via the large articulation between the insert and metallic shell [6, 25]. The utility of this design concept is borne out by low dislocation rates reported in several studies this decade with cementless dual-mobility cups [6–11]. In the current study, there were no dislocations in any subjects. The survivorship for the dual-mobility cup used in this study agrees with previously reported midterm outcomes for this system [26, 27]. In addition, our mean postoperative WOMAC and Merle D’Aubigne scores are comparable to those observed in mid-term follow-up series of cementless dual-mobility cups in primary THR [6, 8, 28].

There have been concerns raised that dual-mobility cups can contribute to increased polyethylene wear at the site of articulation between its convex surface and the metal surface of the acetabular component, as well as due to impingement of the neck on the rim of the retentive polyethylene [7]. However, this is disputed by simulation studies [7] and retrieval analyses of dual-mobility cups [29, 30]. A recent analysis of 35 explanted liners from dual-mobility cups found the wear to be lower than that reported with equivalent cementless liners [30]. Radiographic analysis from the current study provided no indication that liner wear was an issue after a decade in situ.

The cementless triple-taper femoral stem employed in this analysis is the more extensively studied of the components in this combination, though performance data remain limited to survivorship estimates from a pair of publications and registry reports [31–35]. Assaf et al. reported no revisions of the stem in a cohort of 114 THRs followed for 7 years [31], whereas Lee and Evans reported that the subject stem was associated with a cumulative survival rate of 99.1% at 3 years for over 600 consecutive subjects [32]. The National Joint Registry for England, Wales, Northern Ireland, and the Isle of Man reported 98.9% (95% CI, 98.4–99.2) survivorship at 5 years follow-up [33].

There are several potential reasons that account for the reported stability of this femoral stem. The triple-taper stem design concept was created to enhance fixation, reduce subsidence, and improve loading of the proximal femur neck to avoid stress shielding [14, 36]. Triple-taper design has also been noted to result in less periprosthetic bone mineral density loss than straight-type components [14].

Furthermore, the relatively short length of the cementless stem employed here may convey advantages, including greater preservation of bone and optimized proximal load transfer [37]. Compared with standard-length stems, their use has been associated with increased metaphyseal filling [38], decreased intraoperative complications [37, 39], and less thigh pain [39]. The stem also incorporates a titanium plasma spray with hydroxyapatite coating, which may result in improved bone remodeling, subsidence, and migration [40, 41].

Despite being a follow-up of a large cohort of subjects, the study does have some limitations. The study is a retrospective design, which is generally considered to be a lower level of evidence than prospective studies. Another limitation was the use of the Merle d’Aubigne Score, which despite its widespread use, does suffer from well-documented ceiling effects [42–44]. Efforts were made to mitigate these limitations by including the option for a prospective visit beyond 10 years follow-up and including the WOMAC score. Finally, the study was only able to provide follow-up data on 48.6% (244 out of 502) of THRs among the original cohort. The primary reason for this was that 30.3% of participants were deceased. Although this undoubtedly impacts the survivorship analysis, it is an unavoidable consequence of conducting follow-up studies of this duration in a relatively older population.

In conclusion, the subject femoral stem was associated with excellent survivorship and satisfactory functional outcomes at over 10 years follow-up. The study shows that this stem design in combination with a dual-mobility cup offers a safe and effective treatment of subjects requiring THR due to osteoarthritis over a wide range of subject ages.

## Data Availability

The datasets used and/or analyzed during the current study are available from the corresponding author on reasonable request.

## Abbreviations

ODEP: Orthopaedic Data Evaluation Panel
THR: Total hip replacement
WOMAC: Western Ontario and McMaster Universities Osteoarthritis
